# The Interaction between Circulating Cell-Free Mitochondrial DNA and Inflammatory Cytokines in Predicting Human Mental Health Issue Risk in Adolescents: An Explorative Study

**DOI:** 10.3390/biomedicines11030818

**Published:** 2023-03-07

**Authors:** Arto Alatalo, Izaque de Sousa Maciel, Nina Kucháriková, Sweelin Chew, Irene van Kamp, Maria Foraster, Jordi Julvez, Katja M. Kanninen

**Affiliations:** 1 A.I. Virtanen Institute for Molecular Sciences, University of Eastern Finland, 70211 Kuopio, Finland; 2 National Institute for Public Health and the Environment, 3721 MA Bilthoven, The Netherlands; 3 ISGlobal, 08036 Barcelona, Spain; 4 Universitat Pompeu Fabra (UPF), 08005 Barcelona, Spain; 5 CIBER Epidemiología y Salud Pública (CIBEREsp), 28029 Madrid, Spain; 6 PHAGEX Research Group, Blanquerna School of Health Science, Universitat Ramon Llull (URL), 08025 Barcelona, Spain; 7 Clinical and Epidemiological Neuroscience Group (NeuroÈpia), Institut d’ Investigació Sanitària Pere Virgili (IISPV), 43007 Reus, Spain

**Keywords:** mental health, adolescence, mitochondrial DNA, biomarkers, oxidative stress, inflammation

## Abstract

Adolescence is often a challenging time in which psychiatric issues have a strong connection to mental health disorders later in life. The early identification of the problems can reduce the burden of disease. To date, the effective identification of adolescents at risk of developing mental health problems remains understudied. Altogether, the interaction between circulating cell-free mtDNA (ccf-mtDNA) and inflammatory cytokines in adolescents is insufficiently understood regarding experienced mental health difficulties. Our study selected the participants based on the Strength and Difficulty Questionnaire (SDQ) score using the cut-off points of 3 and 18 for the low and the high score groups, respectively. The answers of the SDQ at the age of 12.2–15.7 years contributed to the investigation of (i) whether ccf-mtDNA units are associated with cytokines, and (ii) if an interaction model for predicting risk of mental health issues is observed. We discovered a sex-specific correlation between the screened markers associated with mental health problems in the low and high SDQ score groups among the male participants and in the low SDQ score group among the female participants. The mitochondrial *MT-ND4* and *MT-CO1* genes correlated significantly with interleukin-12p70 (IL-12p70) in males and with monocyte chemoattractant protein-1 (MCP-1) in females. Due to the nature of the explorative study, the studied markers alone did not indicate statistical significance for the prediction of mental health problems. Our analysis provided new insight into potential plasma-based biomarkers to predict mental health issues.

## 1. Introduction

The development of the brain is critical between the ages of 12 and 20 [1]. Due to the rapid development of the central nervous system and cortical control regions, excessive risk-taking behavior and psychiatric issues experienced in early adolescence have a strong link to mental health problems in adult life [2]. Prior to the COVID-19 pandemic, global statistics estimated the general prevalence of diagnosed mental health disorders to cause death or disability in the case of 13.5% of 10-14-year-old (early) adolescents after diagnosis [3]. The pandemic further increased mental health problems due to social and physical restrictions—a study by Graupensperger et al. indicated significant increases in depression symptoms (*p* < 0.01) and loneliness (*p* < 0.001) during the initial phase of the COVID-19 pandemic [4]. Early identification of mental health problems is central to reducing the number of individuals that suffer from mental illness, improving the quality of life of affected individuals, and reducing socioeconomic costs. Approximately 50% of young adults with the long-term NEET (not in education, employment, or training) status have been diagnosed with a psychiatric disorder in adolescence [5]. Hormone levels and the onset age of puberty have been widely studied as contributing factors to brain development between the sexes. Increased testosterone and dehydroepiandrosterone (DHEA) levels in males and estradiol levels in females are associated with physical changes as an indicator of puberty [6]. In particular, the development of psychiatric disorders around the time of the increased sex hormone levels may be sex-specifically implicated with brain development. Furthermore, biological, cognitive, and emotional differences can precede the expression of cognitive problems that are found to vary between males and females [7].

To date, a model combining psychiatric and molecular methods for the effective identification of individuals at heightened risk of developing mental health problems in adolescence does not exist. In psychiatric studies, the SDQ is used as a tool to determine a general score of problem behavior and to generate separate scores for emotional symptoms, conduct problems, hyperactivity, peer relationship problems, and prosocial behavior [8,9]. The low and high SDQ score groups can be categorized using a summary mental health index (the p-factor), which is described as a single-factor indicator that predisposes people to psychopathology [10]. The SDQ total scale is a commonly used general psychopathology measure [11]. A high p-factor score indicates larger life impairment, and poorer developmental histories [10]. Furthermore, the internalizing and externalizing sides of the p-factor measure observable symptoms of anxiety and disruptive behaviors of conduct disorder, respectively [11]. Detailed information on the SDQ scores is described in Materials and Methods.

The immune-inflammatory biomarker alterations are constantly associated with psychiatric conditions and negative clinical outcomes. Recent studies reported blood-based markers, including mitochondrial and cytokine contents, of major depressive disorder, bipolar disorder, and schizophrenia in adults [12,13]. In detail, mitochondria regulate energy production, lipid metabolism, and redox status as a part of maintaining cellular homeostasis. During cellular states of dyshomeostasis, mitochondrial respiration generates increased levels of reactive oxygen species (ROS), a phenomenon known to disturb cellular function and pathological conditions [14,15]. Excessive mitochondrial ROS (mtROS) also damages and fragments mitochondrial DNA (mtDNA) [15,16]. mtDNA has been described as an agonist of the immune system, via damage-associated molecular patterns (DAMPs) where dying cells release endogenous molecules into the extracellular environment [17]. The mtDNA fragments from the damaged mitochondria are released into the cytosol by autophagy. Cell-free mitochondrial DNA (cf-mtDNA) is recognized by the DAMP-specific receptors, including Toll-like receptors (TLRs), leading to the activation of immune cells, and the triggering of an inflammatory reaction [16,18]. Comprehensively, cytokines in plasma have been broadly identified as the prominent diagnostic biomarkers. For example, activated macrophages are a major source of cytokines and inflammatory mediators, of which interleukin-1 (IL-1) is produced early in the activation phase [19,20]. Along with the plasma samples, significant alteration of cytokines IL-1β, IL-6, IL-10, and IL-1RA was detected in the prefrontal cortex of depressed individuals who died by suicide compared with nonpsychiatric controls [21]. A correlation between cf-mtDNA levels and interleukin-4 (IL-4) produced by T-helper 2 (Th2) cells has previously been reported [13].

Cf-mtDNA becomes ccf-mtDNA when entering the extracellular fluids. Studies focusing on ccf-mtDNA unit alterations postulated this phenomenon as a potential indicator and diagnostic tool in early-stage screening and prognosis of mental disorders [13,22,23]. Kageyama et al. showed ccf-mtDNA levels to be significantly reduced in adults suffering from major depressive disorder (MDD) and bipolar disorder (BD) [13]. Similarly, Gonçalves et al. reported higher levels of plasma ccf-mtDNA are associated with later-life depression in individuals over the age of 60 [24]. Conversely, little information is known about the ccf-mtDNA levels in young individuals suffering from a mental disorder or psychological stress. Jeong et al. reported an increased but not significant difference in the ccf-mtDNA levels between the diagnostic groups of BD and the healthy controls at a mean age of 17.0 and 15.5 years old, respectively [25]. Therefore, the consensus on the potential of ccf-mtDNA as a predictive biomarker for mental health dysfunction is controversial. Furthermore, existing studies have not assessed how the sex of participants influences the ccf-mtDNA level.

Based on the reports that (i) childhood trauma is associated with elevated levels of pro-inflammatory cytokines [26], (ii) interleukin-10 (IL-10) is decreased in symptomatic adolescents with BD compared to healthy controls [27], and (iii) the reported nominal correlation between IL-4 and ccf-mtDNA level in adults with MDD [13], we hypothesized that there is a connection between ccf-mtDNA and inflammatory cytokines in adolescents experiencing internalized or externalized mental health outcomes. We investigated whether ccf-mtDNA levels are associated with inflammatory cytokines and can be used as biomarkers for predicting mental health issues in adolescents. Plasma levels of ccf-mtDNA and cytokines were measured in adolescents in the low and high SDQ groups. Here we report a sex-dependent correlation of the ccf-mtDNA unit with inflammatory cytokine levels in relation to the risk for mental health dysfunction. We summarize that the ccf-mtDNA unit is connected to IL-12p70 levels in males and MCP-1 levels in females. Together, these findings indicate novel biomarker panels for mental health risks in adolescents.

## 2. Materials and Methods

### 2.1. Cohort and Samples

Walnuts is a regional, controlled, randomized clinical Spanish cohort originally used for studies on the role of selected fatty acids from walnut intake associated with neuropsychological and physical health. For the current study, the SDQ-based subsamples were selected from the baseline population before the walnut intervention, as described in the next section. The study participants were from Barcelona, Spain, and none of them were taking medication at the time of participating in the study. The peripheral blood collection from all the participants was performed by following a standardized protocol during the school day in 2016–2018 [28]. Fasting samples were not included in the study. Blood was collected to EDTA Plus tubes (BD Biosciences, San Jose, CA, USA), inverted 6 times, and centrifuged at 2500 rcf for 20 min at 4 °C. The plasma layer was separated from the red blood and the buffy coat layers and collected to sterile tubes for storage at −80 °C prior to analyses.

### 2.2. Strengths and Difficulties Questionnaire and Eligibility Criteria

**Figure 1:**
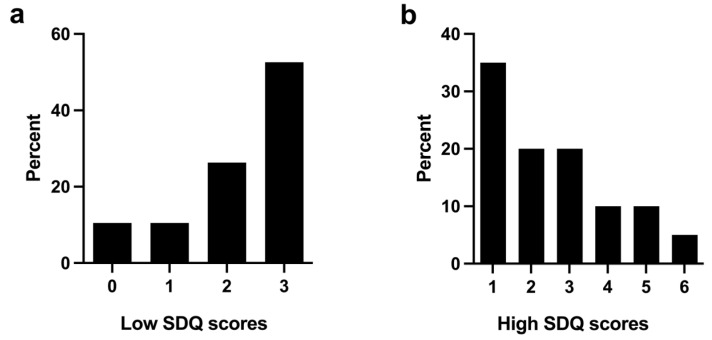
Fraction of SDQ scores among the Walnuts participants among (**a**) low SDQ scores (values of 0–3) and (**b**) high SDQ scores (values of 18–25).

Approximately coincidentally with the collection of blood (Table 1), the participants filled out a self-reported version of the SDQ test. The questionnaire measured a general score of problem behavior and five subscales aimed to assess emotional symptoms, conduct problems, hyperactivity, peer relationship problems, and prosocial behavior [9]. The cut-off point of 18 for the high SDQ score group followed the recoded categorization of the SDQ score [8]. In the case of the low SDQ group, the cut-off point of 3 was selected manually to balance the size of each group (Table 1). The proportions of specific SDQ scores in the low and high groups are shown in Figure 1. The total SDQ score was calculated as a measure psychopathology index, summing hyperactivity, emotional symptoms, conduct problems, and peer problems [8,11]. SDQ is a well-established and widely used index to provide a score ranging from 0–40 (0–14 = low, 15–17 = borderline, 18–40 = high) [8,29].

### 2.3. Extraction and Quantification of Plasma ccf-mtDNA

Circulating cell-free genomic DNA was extracted from 200 μL of plasma by using the QIAamp MinElute Virus Spin Kit (Qiagen, Hilden, Germany) according to the manufacturer’s protocol. The final volume of the eluted ccf-mtDNA was 50 μL, from which the DNA concentration was measured with the NanoDrop ND-1000 spectrophotometer (Thermo Fisher Scientific, Waltham, MA, USA). We focused on two mitochondrial genes that code for separate complexes of the electron transport chain. *ND4* codes for complex I and *CO1* codes for complex IV. TaqMan assays (Thermo Fisher, Carlsbad, CA, USA) were used to quantify two regions of human mtDNA in two replicate runs of qPCR. The *ND4* and *CO1* genes were quantified with TaqMan(R) Gene Expression assays Hs02596876_g1 and Hs02596864_g1, respectively (Table S1). The total reaction volume was 10 μL containing 5.0 μL of Maxima Probe/ROX qPCR Master Mix (Thermo Scientific, Carlsbad, CA, USA), 2.9 μL of the DNA template, 0.5 μL of primer, and 1.6 μL of nuclease-free water (Thermo Scientific, Carlsbad, CA, USA). The genomic DNA template was diluted to a concentration of 7.0 ng/μL with sterile water (Baxter, Mississauga, ON, Canada).

The gene quantification was performed using the StepOnePlus™ Real-Time PCR System (Applied Biosystems™, Carlsbad, CA, USA) for preparing the standard curves and running the extracted gDNA samples. The standard protocol for quantification included initial denaturation and holding steps at 50 °C for 2.0 min and 95 °C for 10 min followed by cycling steps at 95 °C for 15 s and 60 °C for 1.0 min for a total length of 40 cycles. All steps were performed as recommended by the manufacturer.

A standard curve was prepared to calculate and validate the unit of each mitochondrial gene by using commercially manufactured mtDNA plasmids according to the protocol of Applied Biosystems [30]. The plasmids for the standard curve were synthesized commercially (Azenta Life Sciences, Suzhou, China). The inserts of *ND4* (233 nt) and *CO1* (177 nt) genes were ligated into the pUC-GW-Amp plasmids during the manufacturing process (Figure S1). The concentration range of the standard curve was 3.0 × 10^8^ to 3.0 × 10^4^ unit/μL. The coefficient of determination had to be above 0.99 before the curve was approved. The unit level of plasma ccf-mtDNA was based on the standard curves of each quantified mtDNA region. Values are expressed in plasmid units per microliter of plasma.

### 2.4. Cytokine Bead Array

The secreted levels of cytokines in human plasma samples were measured using the Cytometric Bead Array (BD Biosciences, San Jose, CA, USA) along with Human Soluble Protein Master Buffer Kit (BD Biosciences, San Jose, CA, USA) according to the manufacturer´s protocol. IL-12p70 and monocyte chemoattractant protein-1 (MCP-1) were selected from the potential cytokines after screening. Cytokine measurement was performed with the CytoFLEX S Flow Cytometer (Beckman Coulter, Indianapolis, IN, USA). Acquired cytokine data were analyzed in the FCAP ArrayTM v2.0.1 software (Soft flow Inc., New Brighton, MN, USA).

### 2.5. Statistical Analyses

The ccf-mtDNA units were reported as mean ± standard deviation. The *t*-test along with inter-assay coefficients of variation was used to analyze differences between *ND4* and *CO1* unit levels. Unpaired Mann–Whitney U-test was used to compare means between the SDQ-based groups. All tests were two-tailed and a *p*-value ≤ 0.05 indicated a significant difference in means. A nonparametric Spearman’s Rho was used to analyze the correlation between SDQ scores, cytokine levels, and ccf-mtDNA unit. The ccf-mtDNA unit data were tested to confirm the usage of Spearman’s Rho by quantile–quantile plot (QQ plot), which indicated that the data are not following a Gaussian distribution. In the correlation analysis, 0.0 ≤ |r| ≤ 0.2 referred to no correlation, 0.2 < |r| ≤ 0.4 to the low correlation, 0.4 < |r| ≤ 0.6 to the moderate correlation, 0.6 < |r| ≤ 0.8 to the high correlation, and 0.8 < |r| ≤ 1.0 to the very high correlation. The absolute value of effect size was calculated using Cohen’s d. The values were divided into a small effect (d > 0.2), a medium effect (d > 0.5), and a large effect size (d > 0.8) [12]. All analyses were carried out in GraphPad Prism 9.1.1 (GraphPad Software, San Diego, CA, USA) or Microsoft Excel version 2122 (Microsoft, Redmond, WA, USA)

## 3. Results

### 3.1. Ccf-mtDNA Unit Level Does Not Correlate with SDQ Scores or Sex in Adolescents

We first tested the difference in the unit levels between the mitochondrial *CO1* and *ND4* genes and no significant difference was observed (*p* = 0.185). Additionally, to verify a need for two ccf-mtDNA markers, we calculated 33.0% and 37.8% inter-assay coefficients of variation in the unit levels of *ND4* and *CO1* in the cases of the low SDQ score group and the high SDQ score group, respectively.

**Figure 2:**
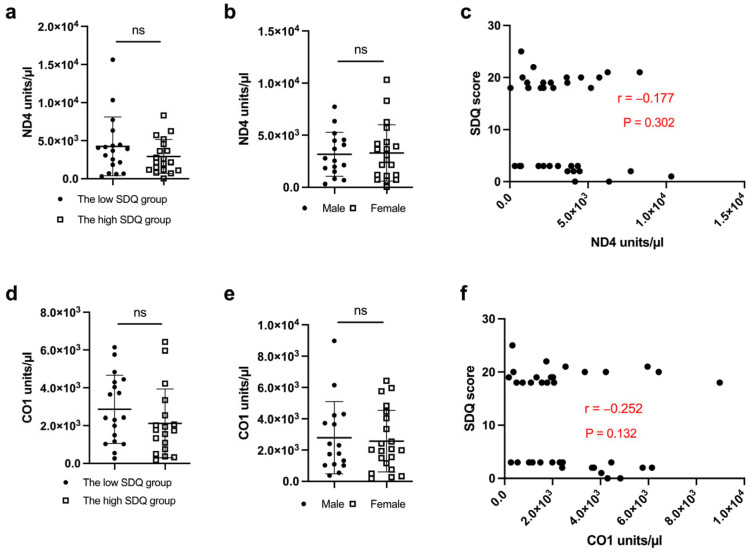
Comparison of plasma ccf-mtDNA unit levels between low and high SDQ groups, including sex-specific differences. (**a**) *ND4* unit level in low (*n* = 18) and high (*n* = 19) SDQ groups, (**b**) *ND4* unit level in males (*n* = 16) and females (*n* = 20), (**c**) interaction of the SDQ score with the level of *ND4* units, (**d**) *CO1* unit level in the low and high SDQ groups (*n* = 18/group), (**e**) *CO1* unit level in males (*n* = 16) and females (*n* = 21), (**f**) interaction of the SDQ score with the level of *CO1* unit. Note: outliers were removed separately from each graph based on the used method. ns: non-significant.

Next, we evaluated the relationship of the self-reported SDQ score and sex with the expression of the mitochondrial *ND4* gene in plasma samples. There was no significant difference in *ND4* levels between the low and high SDQ score groups (Figure 2a) or sexes (Figure 2b). The absolute value of effect size d was 0.424 and 0.047, as shown in Figure 2a,b, respectively. The correlation of *ND4* unit levels and SDQ score did not indicate a significant correlation (Figure 2c; r = −0.177, *p* = 0.302). Subsequently, we analyzed the effects of the SDQ score and sex on the expression of the mitochondrial *CO1* gene. There was no significant difference in *CO1* gene expression between the low and high SDQ groups (Figure 2d) or sexes (Figure 2e). The absolute value of effect size d was 0.326 and 0.109, as shown in Figure 2d,e, respectively. *CO1* unit levels and the SDQ score did not indicate significant correlations (Figure 2f; r = −0.252, *p* = 0.132).

### 3.2. Cytokine Levels Are Not Related to SDQ Scores or Sex in Adolescents

To assess whether cytokines were a potential indicator of the increased risk for mental health disorders in adolescents, we analyzed plasma levels of IL-12p70 and MCP-1. Both cytokines were described as the downstream cytokines of IL-1β in a DAMP-associated inflammation [31,32].

**Figure 3:**
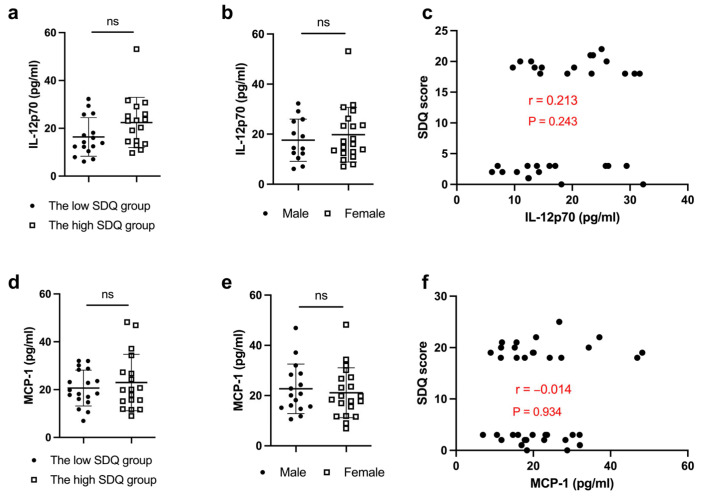
Comparison of plasma cytokine levels between low and high SDQ groups, including sex-specific changes. (**a**) Difference in IL-12p70 expression level between the low (*n* = 16) and high SDQ group (*n* = 17), (**b**) difference in IL-12p70 expression level between male (*n* = 13) and female (*n* = 20), (**c**) interaction of the SDQ score with the level of IL-12p70, (**d**) difference in MCP-1 expression level between the low and high SDQ group (*n* = 18/group), (**e**) difference in MCP-1 expression level between male (*n* = 18) and female (*n* = 20), (**f**) interaction of the SDQ score with the level of MCP-1. Note: outliers were removed separately from each graph based on the used method. ns: non-significant.

The effect of the self-reported SDQ score and sex on the cytokine level of IL-12p70 was studied using the cytokine bead array. There was no significant difference in IL-12p70 levels between the low and high SDQ score groups (Figure 3a), or between males and females (Figure 3b). The absolute value of effect size d was 0.624 and 0.219, as shown in Figure 3a,b, respectively. There was no significant correlation of IL-12p70 concentration and SDQ scores (Figure 3c; r = 0.213, *p* = 0.243). Similarly, MCP-1 levels were not affected by the SDQ score (Figure 3d) or sex (Figure 3e). The absolute value of effect size d was 0.237 and 0.160, as shown in Figure 3e,f, respectively. The correlation of MCP-1 concentration and the SDQ score was not significant (Figure 3f; r = −0.014, *p* = 0.934).

### 3.3. Mitochondrial Genes Correlate with IL-12p70 or MCP-1 in a Sex-Specific Fashion

**Figure 4:**
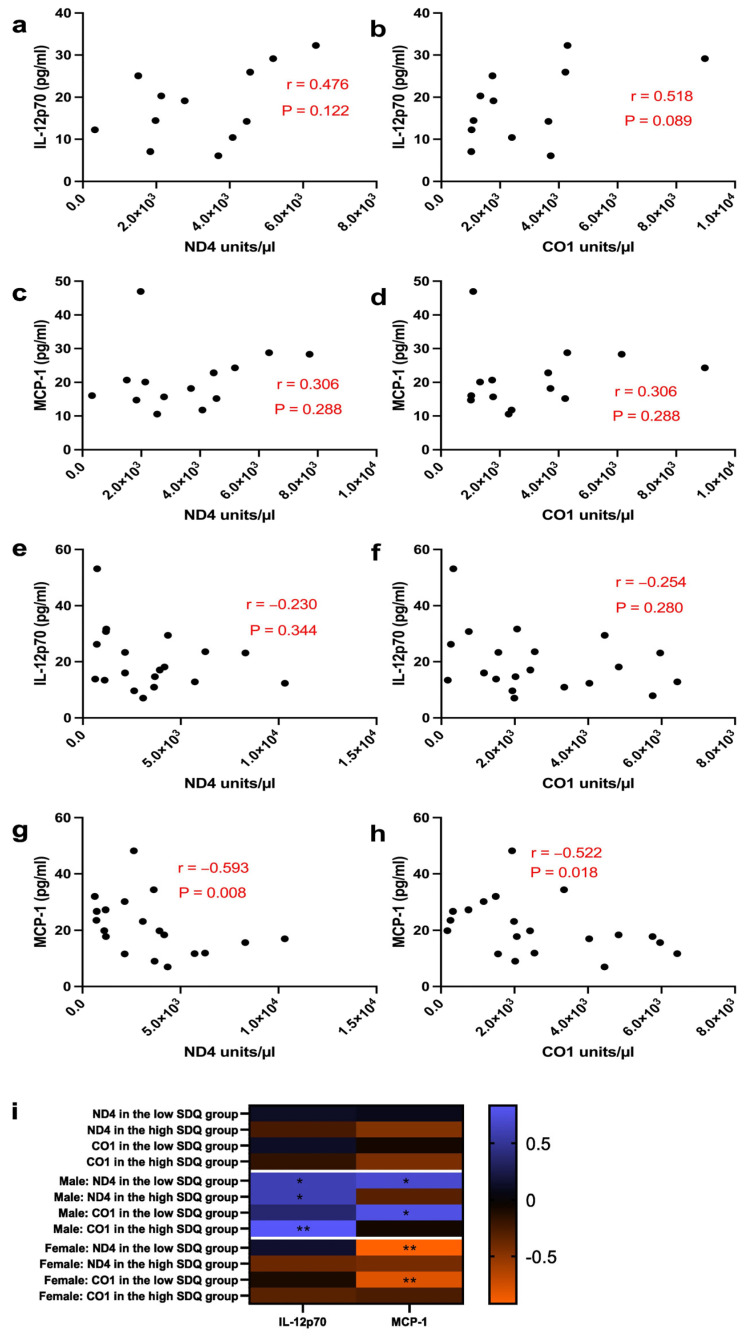
Correlation analysis of cytokine levels and ccf-mtDNA units. (**a**–**d**) Correlation analysis of plasma IL-12p70 and MCP-1 levels with those of *ND4* and *CO1* units in males. (**e**–**h**) Correlation analysis of plasma IL-12p70 and MCP-1 levels with those of *ND4* and *CO1* units in females. (**i**) Clustering heatmap analysis for correlation of the cytokines IL-12p70 and MCP-1 and the genes *ND4* and *CO1* units in all the samples, and separated by sex. (* High correlation 0.6 < |r| ≤ 0.8, ** very high correlation 0.8 < |r| ≤ 1.0.)

We next analyzed the correlation between the ccf-mtDNA markers, *ND4* and *CO1*, and inflammatory cytokines, IL-12p70 and MCP-1, separated by sex. In males, IL-12p70 correlated moderately with both *ND4* (Figure 4a; r = 0.476, *p* = 0.122) and with *CO1* (Figure 4b; r = 0.518, *p* = 0.089). Additionally, there was a low correlation between MCP-1 and *ND4* (Figure 4c; r = 0.306, *p* = 0.288) and *CO1* (Figure 4d; r = 0.306, *p* = 0.288). In females, only a low correlation between IL-12p70 and *ND4* (Figure 4e; r = −0.230, *p* = 0.344) or *CO1* (Figure 4f; r = *−*0.254, *p* = 0.280) was observed. Additionally, we found MCP-1 correlated moderately with both *ND4* (Figure 4g; r = −0.593, *p* = 0.008) and *CO1* (Figure 4h; r = −0.522, *p* = 0.018) in females.

Subsequently, we tested the interaction of the mitochondrial genes and inflammatory cytokines as described by Kageyama et al. [13]. The correlation between the ccf-mtDNA units and cytokine levels separated by sex were assessed by the Spearman’s Rho (Figure 4i). In male subjects, a very high positive correlation of both mitochondrial *ND4* (r = 0.600, *p* = 0.242) and *CO1* (r = 0.829, *p* = 0.058) with IL-12p70 were observed in the high SDQ score group. Additionally, a high positive correlation of both mitochondrial *ND4* (r = 0.691, *p* = 0.069) and *CO1* (r = 0.762, *p* = 0.037) with hMCP-1 was observed in males of the low SDQ score group. In females, a very high negative correlation of both mitochondrial *ND4* (r = −0.917, *p* = 0.001) and *CO1* (r = −0.800, *p* = 0.014) with MCP-1 was observed in the low SDQ score group. No significant correlations between mitochondrial genes and cytokines were observed in females with a high SDQ score. Furthermore, IL-12p70 was not significantly correlated to the mitochondrial genes of females in either the low or high SDQ score group.

## 4. Discussion

Here, we measured the correlation between ccf-mtDNA and cytokine markers, implicating a potential connection where the extracellular mtDNA may trigger an immune-inflammatory reaction in subjects at risk of psychopathology. The results indicate, on the whole, the existence of a specific correlation between the levels of ccf-mtDNA markers (*ND4* and *CO1*) and inflammatory markers (IL-12p70 and MCP-1) to predict an increased risk for mental health issues compared with low-risk controls. Together, these findings indicate proposing novel biomarker panels for mental health risks in adolescents. Conversely, our study did not unveil the potential of using single ccf-mtDNAs or cytokines as predictive markers. Next, it is important to complete our predictive model by analyzing the role of other potential cytokine markers in the inflammatory pathway. To the best of our knowledge, this study reports for the first time how the risk of mental health issues in adolescents is connected to plasma ccf-mtDNA unit level, an indicator of oxidative stress, and cytokine levels, indicators of inflammation. Along with the analysis of the adolescent samples, we also provided insight into the inflammation process triggered by ccf-mtDNA, which is not affected by aging or chronic diseases. Our results suggest that inflammatory cytokine changes can potentially be associated with ccf-mtDNA markers in adolescents, but the association is sex-specific.

The genes of mitochondrial complex I (ND1-ND6) are the most studied genes of ccf-mtDNA associated with mental health disorders [12,13,25,33]. To date, little research has been conducted on the role of the genes encoding other mitochondrial complexes. Therefore, we decided to also perform quantitative analysis for the *CO1* gene, part of complex IV, to discover whether the location of the gene can affect the results. The calculated inter-assay coefficient of variation in the ccf-mtDNA unit level was above 30% to indicate the importance of measuring the unit level of both genes. Conversely, the *t*-test did not indicate significant differences between the unit levels of *CO1* and *ND4* (*p* = 0.185). However, the results from the coefficient of variation test provided a reason to test the expression of both genes in the plasma samples. Our results did not indicate significant variation in the unit levels of *ND4* and *CO1* between sexes or between the SDQ score groups (Figure 2), which emphasizes the reliability of the process. It also suggests that endogenous differences do not impact the quantification of the studied ccf-mtDNA genes. Additionally, a relatively small sample number affected the effect size of this study. The absolute value of effect size d was 0.33–0.42 between the low SDQ and high SDQ score groups in the quantification of mtDNA unit levels.

Previously, Lindqvist et al. and Kageyama et al. reported ccf-mtDNA units among adults with a diagnosed mental health disorder when compared to healthy controls. However, the Kageyama et al. paper reported a decrease, while the Lindqvist et al. study showed an increase in ccf-mtDNA level in individuals suffering from a mental disorder [12,13]. Moreover, Jeong et al., studied serum ccf-mtDNA unit levels in adolescents with a diagnosis of BD without finding significant differences when compared to healthy individuals [25]. Their findings on adolescents with an actual mental health disorder are in line with the results of our study, which indicated that ccf-mtDNA unit levels are not significantly altered in adolescents that are at risk of mental problems, as indicated by the results of the self-reported SDQ test. Based on these findings, we postulated that the SDQ score has utility in studies that focus on the risk of mental health, prior to an actual disease diagnosis. Along with our studies, the SDQ scores have also been used in earlier biomarker analyses to assess the risk of emotional and behavioral difficulties among children or adolescents [34,35]. Goodman and Goodman showed in their studies that children with higher total difficulty SDQ scores have greater psychopathology, which can be predictive of a disorder status three years later [36].

We investigated whether plasma ccf-mtDNA could trigger an inflammatory response, which may occur via the activated Toll-like receptor 9 (TLR9) [16]. Our study focused on measuring two pro-inflammatory cytokines (IL-12p70 and MCP-1) in the plasma samples. The heterodimeric IL-12p70 cytokine connects innate and adaptive immunity to stimulate T and natural killer (NK) cells [37]. Furthermore, interferon gamma (IFN-γ) serves as a signal to activate IL-12p70 production followed by the formation of a positive-feedback loop with IFN-γ [38,39]. On the other hand, IL-12p70 can be induced by pathogenic DNA [40]. Based on our results, both IL-12p70 and MCP-1 levels are associated with an increased risk of mental health issues, which has previously been highly understudied in the plasma samples. In addition, earlier studies indicated that excessive MCP-1 production can potentially favor Th2-specific immune responses [31]. When analyzing the plasma-based cytokines, we did not observe statistically significant differences between the low and high SDQ groups in the levels of IL-12p70 or MCP-1 in adolescents (Figure 3a,d). Additionally, plasma IL-12p70 and MCP-1 levels did not correlate significantly with the self-reported SDQ scores and, therefore, are not, on their own, sufficient biomarkers to predict an increased risk for mental health problems.

At this point, no studies have been conducted to point out straightforward correlations between ccf-mtDNA genes and IL-12p70 or MCP-1 in plasma. Nishimoto et al. indicated with animal studies that activation of the cfDNA-TLR9 pathway increases the expression of MCP-1 in macrophages [41]. A similar result was observed in the case of IL-12 production in dendritic cells [42]. Interestingly, our study revealed a sex-related difference in the results. We observed a clear effect of sex on the interaction of oxidative (ccf-mtDNA) and inflammatory (cytokine) markers. The direction of correlation was positive among the boys and negative among the girls. Surprisingly, the correlation was moderately high between the ccf-mtDNA markers and IL-12p70 among the boys (Figure 4a,b) but not among the girls (Figure 4e,f). Opposite results were found in the correlation analysis of MCP-1 with the ccf-mtDNA markers where the correlation was moderately high among the girls (Figure 4g,h) but not among boys (Figure 4c,d). When analyzing only the effect of the high SDQ score on the results, the units of *ND4* and *CO1* were highly or very highly positively correlated with the level of IL-12p70 but not with MCP-1 in male adolescents (Figure 4i). Among the female participants in the high SDQ score group, the correlation was negative and less significant between *ND4* or *CO1* and IL-12p70 or MCP-1 (Figure 4i) than in the male group. One possible explanation for the different results could be the contribution of the sex hormones and the stage of puberty. The mean ages for the blood sampling were 13.7 and 14.0 years for the male and female participants, respectively. In the case of the female participants, 85.7% reported having their first periods before taking the SDQ test, while in males, only 16.7% reported having started to develop facial hair before taking the SDQ test. Based on these reports, it is possible that the female participants were at a more advanced puberty stage at the time of testing, although their biological age was similar to the male participants. The sex-specific effect from hormones associated with the divergent oxidative stress and inflammatory pathway profiles between the male and female groups cannot be ruled out. Sex differences have previously been associated with mitochondrial- or mtDNA-related processes such as antioxidant defense, reactive oxygen species production, and immune response [43,44]. Pubertal hormonal (testosterone or estradiol) activity begins to increase cortical volumes earlier in girls than in boys [45]. Additionally, there are sex-specific differences in oxidative markers such as 8-oxoguanine (8-oxoG), GSH, and H_2_O_2_ associated with reactive oxygen species [46,47,48]. The role of pubertal hormones is most likely one of the main reasons why we observed a positive correlation between ccf-mtDNA markers and cytokines among boys and a negative correlation among girls (Figure 4). The hypothesis about the effect of sex hormones is supported by Carrascosa et al. in their Spanish longitudinal growth study, where the calculated mean onset age for the pubertal growth spurt was 11.97–12.96 years in boys and 9.91–10.90 years in girls [49]. Thus far, there have been very limited numbers of sex-specific studies to emphasize the difference of sex in the unit level of ccf-mtDNA associated with the risk of mental health issues or mental disorders in adolescents. However, Trumpff et al. reported higher serum ccf-mtDNA levels in middle-aged men than women in response to stress [33].

Despite the finding of a sex-specific significant correlation between biomarkers, a relatively small number of samples (*n* = 18–21 per group) together with unequal numbers of samples in the low and high SDQ groups and relatively low effect size are potential limitations of this explorative study. Therefore, we cannot exclude that the used n number could influence the statistical power of the analyses and conclusions. Kageyama et al. used 87–107 samples per group and Jeong et al. analyzed 105 samples [13,25]. Lindqvist et al. analyzed 37 plasma samples per group in their study with an effect size d of 2.55–4.01 [12]. Additionally, the sample preparation method including the duration and speed of centrifugation for plasma are described as affecting the ccf-mtDNA specificity by isolating different forms of ccf-mtDNA [23]. In our study, the same preparation method was used for all the plasma samples. We also quantified the nuclear DNA (nDNA) housekeeping gene levels in our plasma samples in order to eliminate possible contamination of different DNA material other than mtDNA. Furthermore, this study did not account for confounding factors such as mechanical tissue injuries or exercising, which have previously been linked to immediate and significant increases in ccf-mtDNA [50,51]. For example, an increase in the ccf-mtDNA level immediately after physical exercise was observed by Stawski et al. [52] and Beiter et al. [53] in men. Further studies could also focus on assessing the relationship between physical exercise and ccf-mtDNA in adolescents. Because the regular psychotropic medication is known to affect ccf-mtDNA levels [54], the participants of this study did not take any medication before donating the blood sample.

Our data about the interaction of the quantified ccf-mtDNA markers and the screened inflammatory cytokines (Figure 4) give, for the first time, an indicator of a potential pathway model to predict the risk of mental health issues in adolescents. The suggested framework for the interaction model is sex-specific, and it proposes that ccf-mtDNA triggers TLR9 followed by the activation of pro-inflammatory cytokines. However, based on the analyzed data, the correlation between ccf-mtDNA and cytokine markers is positive among boys and negative among girls. We believe that the observed sex-dependent changes are related to sex hormones and the stage of puberty. More studies are needed to verify the model and its function as a potential diagnostic tool.

## 5. Conclusions

In conclusion, our explorative study provides a new insight into potential plasma-based biomarkers to predict increased risk for mental health problems in children or adolescents. Our suggested model for connecting oxidative stress markers with pro-inflammatory cytokines is based on a relatively small number of samples used in this study. Further studies with a greater n number and specific information about the health issues and other lifestyle factors of participants are needed to validate the correlation analyses. A larger n number would also empower the effect size, which is smaller in our study than in other mtDNA-focused mental health issue studies. In addition, we were not able to assess the effect of sex-specific hormone levels on the observed results. However, studying blood-based markers in adolescents, without chronic diseases or long-lasting medication, which could affect the results, can be beneficial to limit the variation often observed with samples derived from adults. Further molecular level studies are needed to verify our suggested model, and to assess the role of sex hormones (testosterone or estradiol) in relation to ccf-mtDNA and cytokine levels in adolescents. We believe that our current findings can be used as a baseline for bigger cohort studies to set out detailed and comprehensive diagnostic models between oxidative and immune-inflammatory biomarkers associated with mental health issues.

## Figures and Tables

**Table 1:** Demographic information and the range of the SDQ score, the execution age of SDQ and blood tests, and the sex distribution of study subjects.

Group	Score	Age of SDQ Test (Years)		Age of Blood Test (Years)		Samples		
		Mean	SD	Mean	SD	*n*	Male	Female
Low SDQ	0–3	13.48	0.80	13.51	0.81	19	10	9
High SDQ	18–25	14.11	1.07	14.15	1.15	20	8	12

## Data Availability

The data presented in this study are available on request from the Institutional Data Access/Ethics officer. The data are not publicly available due to the consent of personal information of children, which needs to be kept confidential.

## Supplementary Materials

The following supporting information can be downloaded at: https://www.mdpi.com/article/10.3390/biomedicines11030818/s1, Figure S1: Sequence analysis of the copy number plasmids; Table S1: Information of TaqMan Gene Expression assays.
