# Supplementary material for: The Interaction between Circulating Cell-Free Mitochondrial DNA and Inflammatory Cytokines in Predicting Human Mental Health Issue Risk in Adolescents: An Explorative Study
Source: Biomedicines. 2023 Mar 7;11(3):818. doi: 10.3390/biomedicines11030818 (PMC10045177; doi:10.3390/biomedicines11030818)

Figure S1. Sequence Analysis of the copy number plasmids

5'EcoRI highlighted in Yellow
3'EcoRI highlighted in Cyan

MT-ND4: A 69 T 61 C 76 G 27 | GC%: 44.21% | Length: 233

GAATTCTAGGCTCCCTTCCCCTACTCATCGCACTAATTTACACTCACAACACCCTAGGCTCACTAAACATTCTACTACTCACTCTCACTGCCCAA
ACTATCAAACTCCTGAGCCAACAACTTAATATGACTAGCTTACACAATAGCTTTTATAGTAAAGATACCTCTTTACGGACTCCACTTATGACTCC
AAAGCCCATGTCGAAGCCCCCATCGCTGGGTCAGAATTC

GC Content

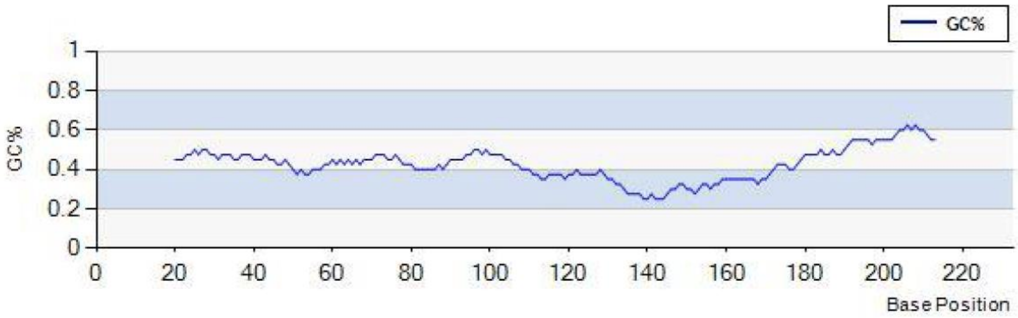

MT-CO1: A 44 T 41 C 68 G 24 | GC%: 51.98% | Length: 177

GAATTCCCAATACCAAACGCCCCTCTTCGTCTGATCCGTCCTAATCACAGCAGTCCTACTTCTCCTATCTCTCCCAGTCCTAGCTGCTGGCATCA
ATACTACTAACAGACCGCAACCTCAACACCACCTTCTTCGACCCCGCCGGAGGAGGAGACCCCATTCTATACCAGAATTC

GC Content

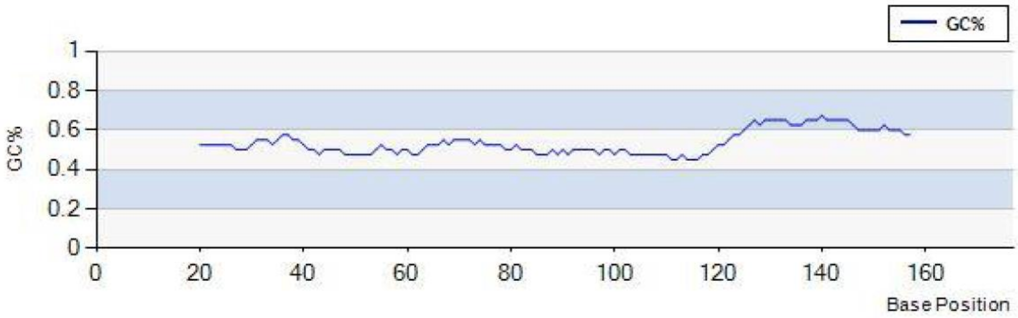

Supplement: Supplementary file 1 [file biomedicines-11-00818-s001.zip › Supplementary Figure S1.pdf]
