# Supplementary material for: The Interaction between Circulating Cell-Free Mitochondrial DNA and Inflammatory Cytokines in Predicting Human Mental Health Issue Risk in Adolescents: An Explorative Study
Source: Biomedicines. 2023 Mar 7;11(3):818. doi: 10.3390/biomedicines11030818 (PMC10045177; doi:10.3390/biomedicines11030818)
Supplement: Supplementary file 1 [file biomedicines-11-00818-s001.zip › Supplementary Table S1.pdf]

Table S1. Information of Taqman Gene Expression assays

Gene	Assay ID	Context Sequences (5′→3′)	Amplicon length
mt-ND4	Hs02596876_g1	AACTCCTGAGCCAACAACTTAATAT	150 nt
mt-CO1	Hs02596864_g1	GTCCTAGCTGCTGGCATCACTATAC	94 nt
